# Metagenomic Sequencing of HIV-1 in the Blood and Female Genital Tract Reveals Little Quasispecies Diversity during Acute Infection

**DOI:** 10.1128/JVI.00804-18

**Published:** 2019-01-04

**Authors:** Anne Piantadosi, Catherine A. Freije, Christina Gosmann, Simon Ye, Daniel Park, Stephen F. Schaffner, Damien C. Tully, Todd M. Allen, Krista L. Dong, Pardis C. Sabeti, Douglas S. Kwon

**Affiliations:** a Division of Infectious Diseases, Massachusetts General Hospital, Boston, Massachusetts, USA; b Broad Institute, Cambridge, Massachusetts, USA; c Harvard Medical School, Boston, Massachusetts, USA; d Ph.D. Program in Virology, Division of Medical Sciences, Harvard University, Boston, Massachusetts, USA; e Ragon Institute of MGH, MIT, and Harvard, Cambridge, Massachusetts, USA; f Harvard-MIT Program of Health Sciences and Technology, Cambridge, Massachusetts, USA; g FAS Center for Systems Biology, Department of Organismic and Evolutionary Biology, Harvard University, Cambridge, Massachusetts, USA; h Department of Immunology and Infectious Disease, Harvard T. H. Chan School of Public Health, Boston, Massachusetts, USA; i Howard Hughes Medical Institute, Chevy Chase, Maryland, USA; Icahn School of Medicine at Mount Sinai

**Keywords:** bottleneck, female genital tract, human immunodeficiency virus, metagenomic

## Abstract

Heterosexual transmission of human immunodeficiency virus type 1 (HIV-1) is associated with a significant bottleneck in the viral quasispecies population, yet the timing of that bottleneck is poorly understood. We characterized HIV-1 diversity in the blood and female genital tract (FGT) within 2 weeks after detection of infection in three women enrolled in a unique prospective cohort in South Africa. We assembled full-length HIV-1 genomes from matched cervicovaginal lavage (CVL) samples and plasma. Deep sequencing allowed us to identify intrahost single-nucleotide variants (iSNVs) and to characterize within-sample HIV-1 diversity. Our results demonstrated very little HIV-1 diversity in the FGT and plasma by the time viremia was detectable. Within each subject, the consensus HIV-1 sequences were identical in plasma and CVL fluid. No iSNV was present at >6% frequency. One subject had 77 low-frequency iSNVs across both CVL fluid and plasma, another subject had 14 iSNVs in only CVL fluid from the earliest time point, and the third subject had no iSNVs in CVL fluid or plasma. Overall, the small amount of diversity that we detected was greater in the FGT than in plasma and declined over the first 2 weeks after viremia was detectable, compatible with a very early HIV-1 transmission bottleneck. To our knowledge, our study represents the earliest genomic analysis of HIV-1 in the FGT after transmission. Further, the use of metagenomic sequencing allowed us to characterize other organisms in the FGT, including commensal bacteria and sexually transmitted infections, highlighting the utility of the method to sequence both HIV-1 and its metagenomic environment.

**IMPORTANCE** Due to error-prone replication, HIV-1 generates a diverse population of viruses within a chronically infected individual. When HIV-1 is transmitted to a new individual, one or a few viruses establish the new infection, leading to a genetic bottleneck in the virus population. Understanding the timing and nature of this bottleneck may provide insight into HIV-1 vaccine design and other preventative strategies. We examined the HIV-1 population in three women enrolled in a unique prospective cohort in South Africa who were followed closely during the earliest stages of HIV-1 infection. We found very little HIV-1 diversity in the blood and female genital tract during the first 2 weeks after virus was detected in the bloodstream. These results are compatible with a very early HIV-1 population bottleneck, suggesting the need to study the HIV-1 population in the female genital tract before virus is detectable in the bloodstream.

## Abstract (precis)

Due to error-prone replication, HIV-1 generates a diverse population of viruses within a chronically infected individual. When HIV-1 is transmitted to a new individual, one or a few viruses establish the new infection, leading to a genetic bottleneck in the virus population. Understanding the timing and nature of this bottleneck may provide insight into HIV-1 vaccine design and other preventative strategies. We examined the HIV-1 population in three women enrolled in a unique prospective cohort in South Africa who were followed closely during the earliest stages of HIV-1 infection. We found very little HIV-1 diversity in the blood and female genital tract during the first 2 weeks after virus was detected in the bloodstream. These results are compatible with a very early HIV-1 population bottleneck, suggesting the need to study the HIV-1 population in the female genital tract before virus is detectable in the bloodstream.

## INTRODUCTION

Heterosexual transmission of human immunodeficiency virus type 1 (HIV-1) is associated with a significant bottleneck in the viral population. Typically, a transmitting partner with chronic HIV-1 infection harbors a diverse viral quasispecies, which is reduced to only one or a few viral variants after transmission to the recipient partner. Prior studies investigating the HIV-1 transmission bottleneck, which mostly examined HIV-1 in the plasma 1 to 3 months after infection, have demonstrated that sexually acquired infection with HIV-1 is established by a single transmitted/founder (T/F) virus in approximately 60% to 80% of individuals (1–7). However, some women acquire a more heterogeneous viral population in the plasma (8) and in the female genital tract (FGT) (9), and a recent study demonstrated greater HIV-1 *env* diversity in the FGT than in blood within the first 3 months after infection (10). Understanding the timing and location of the HIV-1 transmission bottleneck, as well as factors that contribute to it, may provide critical insight into the design of an HIV-1 vaccine and other preventative strategies.

Multiple factors likely contribute to the HIV-1 transmission bottleneck during male-to-female transmission, including compartmentalization within the male genital tract prior to transmission (11). The FGT is also believed to contribute to the HIV-1 transmission bottleneck by providing a mucosal barrier and a limited number of target cells for HIV-1 infection (11). When these factors are disrupted by sexually transmitted infections (STIs) or hormonal contraceptive use, women can acquire a more diverse HIV-1 population (12, 13). The FGT has also been described as supporting compartmentalized evolution of the HIV-1 population throughout infection (14–16). However, relatively little is known about viral populations present in the FGT during the earliest stages of acute infection.

We evaluated very early viral diversity and the HIV-1 transmission bottleneck in the FGT in three subjects from a unique prospective cohort of South African women with hyperacute HIV-1 infection in whom infection was detected prior to the time of peak viral load (17–19). We performed metagenomic sequencing of RNA extracted from plasma and cell-free cervicovaginal lavage (CVL) samples and examined HIV-1 quasispecies present in the blood and FGT during the first 2 weeks after detection of viremia. This approach allowed quantification of HIV-1 variants and assessment of other organisms present in the FGT.

## RESULTS

The Females Rising through Education, Support and Health (FRESH) study enrolls HIV-negative women near Durban, South Africa, and provides a unique opportunity to study HIV-1 infection within days of viremia being detectable (18–20). Women are screened for HIV-1 infection by fingerstick testing every 3 or 4 days, and HIV-1 incidence in the cohort is 8.2 per 100 person-years (19). After detection of infection, women return for weekly collection of blood and FGT samples. Given this frequency of close follow-up, HIV in the FGT is assessed with CVL samples rather than invasive tissue samples; this approach has previously been used to study HIV-1 populations in the FGT (15).

**FIG 1:**
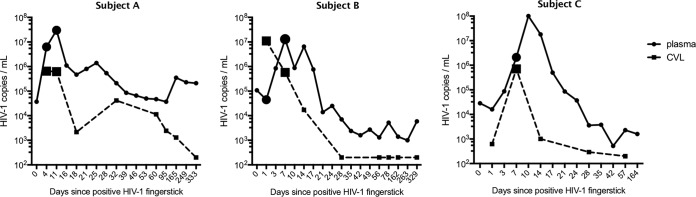
Viral load patterns in plasma and CVL fluid. The plots indicate the HIV-1 load in plasma (solid lines) and the HIV-1 RNA quantification in CVL fluid (dashed lines) for the 3 subjects in this study over time. Viral loads in plasma are not directly comparable to viral loads in CVL fluid due to differences in specimen collection, processing and HIV-1 quantification. Samples used for sequencing in this study are indicated by large circles (plasma) and squares (CVL fluid).

**FIG 2:**
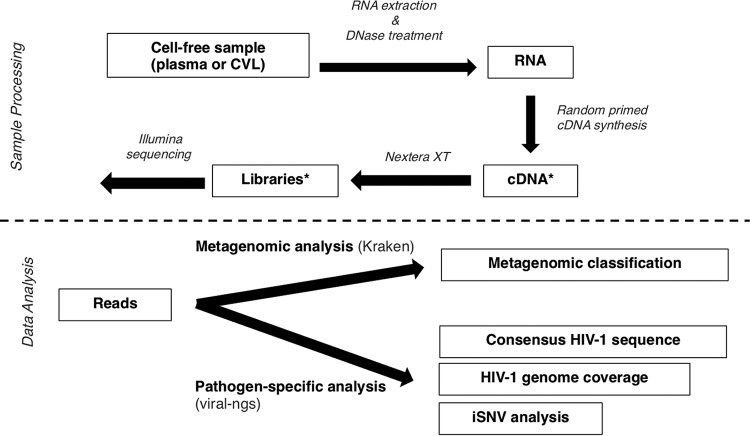
Metagenomic sequencing and analysis approach. The schematics indicate laboratory sequencing methods, metagenomic analysis of microbial content, and HIV-1-specific analysis. The asterisks mark steps in which two independent preparations were performed to ensure reproducibility.

We obtained paired plasma and CVL samples from three subjects diagnosed with HIV-1 infection in Feibig stage I (41). In all three subjects, we included paired plasma and CVL samples from the time of peak HIV-1 load in CVL fluid. This occurred on the fourth day after a positive HIV-1 fingerstick (day 4) for subject A, day 1 for subject B, and day 7 for subject C (Fig. 1 and Table 1). We also included paired samples from the time of peak viral load in plasma for subjects A (day 11) and B (day 7); for subject C, we did not include samples from the time of peak viral load in plasma because it was only 3 days later than the earlier sample. All samples were collected prior to the initiation of antiretroviral therapy. We sequenced a total of 40 million to 250 million RNA reads per sample and performed both HIV-1-specific analysis and metagenomic classification (Fig. 2). As expected, given the unbiased nature of metagenomic sequencing, plasma samples contained relatively few HIV-1 reads (mean, 1.5%) and had a mean of 48.5% human reads. CVL samples had a mean of 0.3% HIV-1 reads and 7.9% human reads.

**TABLE 1:** Summary of samples, HIV-1 levels, and sequencing depths^*a*^

Subject	Sample name	Time point (no. of days since fingerstick)		Plasma HIV-1 load (copies/ml)	CD4 count [cells/mm^3^ (%)]	HIV-1 RNA copies/μl		HIV-1 sequencing depth (mean)	
		First positive	Last negative			Plasma	CVL	Plasma	CVL
A	D4	4	7	6,300,000	306 (30)	382,500	79,750	2,735	192
	D11	11	14	30,000,000	217 (23)	538,500	72,550	1,835	338
B	D1	1	5	445,000	432 (40)	5,686	251,900	37	2,328
	D7	7	12	13,000,000	304 (41)	50,630	56,150	50	206
C	D7	7	11	100,000,000	208 (50)	1,330,000	64,100	1,904	424

a Plasma and CVL samples were selected during the first 2 weeks that viremia was detectable. For each sample, both the number of days since the first positive fingerstick test for HIV-1 and the number of days since the last negative fingerstick test are listed. Clinical measurements of the HIV-1 load in plasma and the CD4 count are shown, as well as the HIV-1 quantification in RNA extracted from both plasma and CVL using qRT-PCR. The mean depth of HIV-1 genome sequencing for each sample is shown, and the sequencing depth across the HIV-1 genome is shown in Fig. 4.

### HIV-1 consensus genomes.

**FIG 3:**
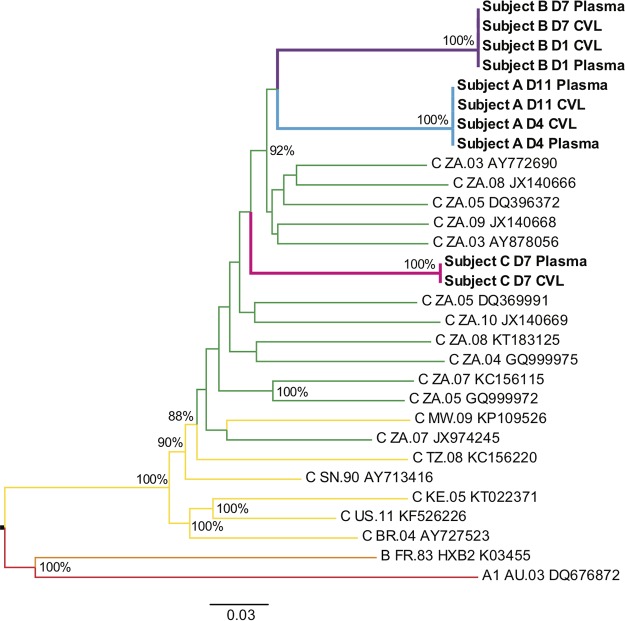
Phylogenetic tree of HIV-1 consensus sequences from each sample. Sequences from subject A are labeled in blue, those from subject B are labeled in purple, and those from subject C are labeled in magenta. Reference subtype C sequences from South Africa are labeled in green, other subtype C sequences are labeled in yellow, one subtype B sequence is labeled in orange, and one subtype A sequence is labeled in red. Sequence names for samples in this study indicate the subject, day of sampling, and sample type. Reference sequences are named by subtype, country of origin, year, and GenBank accession number. Nodes with at least 80% support (out of 1,000 bootstraps) are labeled with the bootstrap values.

Although the HIV-1 reads represented a small proportion of the total reads obtained by metagenomic sequencing, we were able to assemble a consensus HIV-1 genome sequence from each sample. The depth of HIV-1 genome coverage based on unique reads was at least 50× in all but one sample and was at least 1,000× in the four samples with the highest HIV-1 RNA content (Table 1). HIV-1 genomes from all three subjects belonged to subtype C (Fig. 3), the most common HIV-1 subtype in South Africa. The HIV-1 consensus genomes were distinct between different subjects. However, within each subject, the HIV-1 consensus genomes from all the samples were identical: there was no difference between plasma and CVL fluid or between the first and second time points examined. As described below, the consensus genomes represented the vast majority of viruses sequenced, indicating an overall similarity of the HIV-1 populations between the two compartments and little change during the first 2 weeks that viremia was detectable.

### Within-sample HIV-1 single-nucleotide variants.

**FIG 4:**
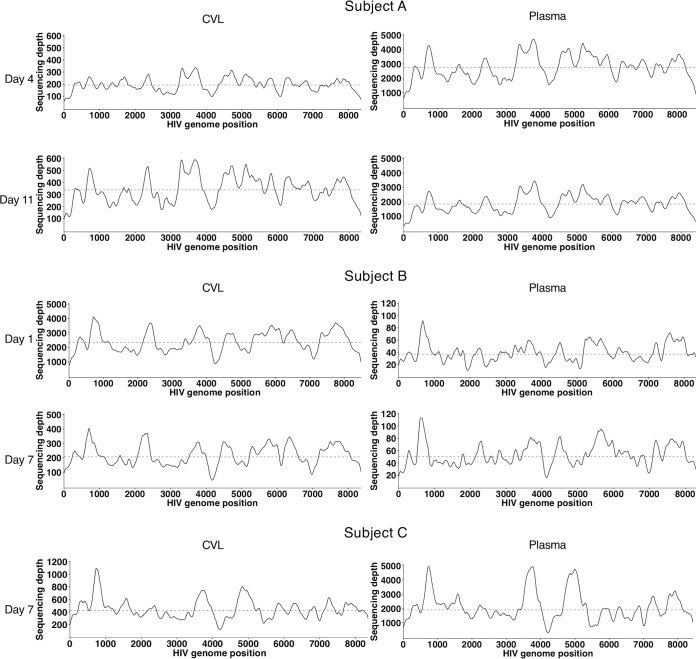
HIV-1 genome coverage for each sample. The plots indicate the sequencing depth across the HIV-1 genome for each sample, which was calculated as a sliding average with a bin width of 100 nt and a sliding window of 10 nt. The coverage represents the sum across both independently prepared sequencing libraries. The dashed lines represent the mean coverage.

In order to characterize the diversity of the HIV-1 quasispecies in each sample, we first mapped all unique HIV-1 reads within each sample to the consensus sequence from that sample. Because our unbiased sequencing approach generated cDNA fragments with unique start and end positions prior to low-cycle library amplification, we were able to remove PCR duplicates by collapsing reads with the same start and end positions to their consensus. We calculated the depth of HIV-1 genome coverage based on unique reads; correspondingly, samples with higher HIV-1 RNA content yielded higher coverage (Table 1). In all the samples, we achieved robust coverage across the HIV-1 genome (Fig. 4), with some variation in depth, likely due to known biases in random-hexamer priming and library construction (21).

We identified within-sample HIV intrahost single-nucleotide variants (iSNVs) using V-Phaser 2 (22). In order to distinguish iSNVs from errors introduced during library construction (including reverse transcription to cDNA) and sequencing, we sequenced duplicate libraries that had been independently constructed from RNA from each sample (Fig. 2). Our stringent parameters for reporting an iSNV required that it be found in both libraries, with at least 0.5% frequency overall, and without substantial strand bias.

**FIG 5:**
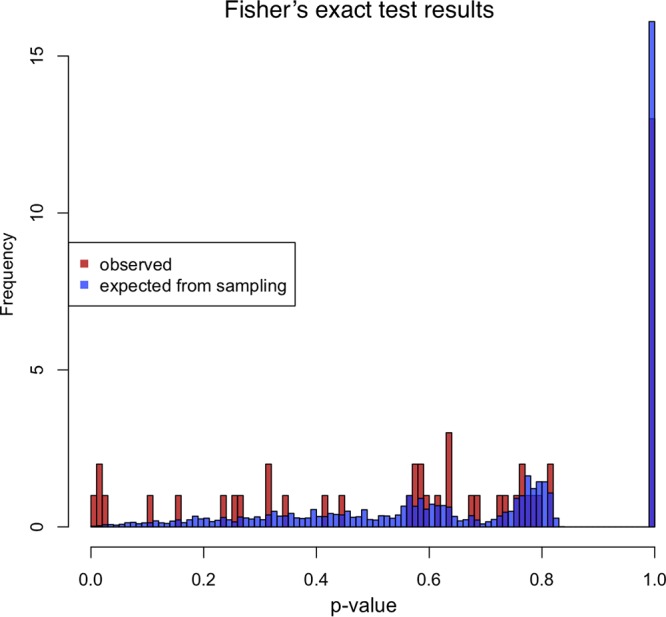
Comparison of SGA and metagenomic sequencing for iSNV detection. For each iSNV in the validation sample, Fisher’s exact test was used to compare the frequency of the iSNV detected by SGA with the frequency of the iSNV detected by metagenomic sequencing. The plot shows a frequency distribution of the resulting *P* values for the observed comparisons, as well as *P* values for simulations performed to assess whether the differences between the two methods were more than could be expected from sampling variance.

We validated this approach by performing metagenomic sequencing on a plasma sample that was previously studied using single-genome amplification (SGA) and 454 sequencing (2). Our metagenomic sequencing and analysis pipeline recovered 90% (44/49) of the previously identified iSNVs (see Table S1 in the supplemental material) and produced no false positives. These results were consistent with expectations from simulations performed to assess whether the differences between the two methods were more than could be expected from sampling variance (Fig. 5). Upon further investigation, we found that the remaining 5 iSNVs were present in our sequencing reads but did not pass our stringent variant-calling filters. We nevertheless maintained our stringent filters for iSNV identification, sacrificing some sensitivity for true iSNVs at low sequencing depth in order to avoid false detection of iSNVs at high sequencing depth.

We then evaluated iSNVs in samples from the three subjects in the study. A full description of the iSNVs identified is provided in Table S2 in the supplemental material, including the iSNV position, linkage with other iSNVs, consensus and variant alleles, overall frequency, and frequency within each of the two duplicate sequencing libraries. Overall, we observed relatively little HIV-1 diversity in these samples from acute infection. Most samples contained few iSNVs, and no iSNV was present at greater than 6% frequency.

**FIG 6:**
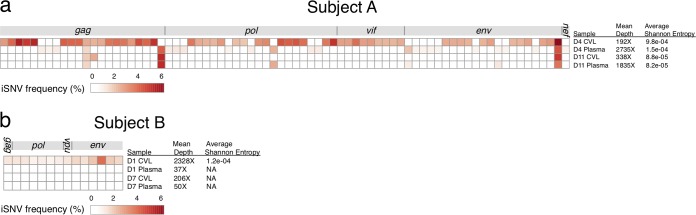
Frequencies of iSNVs in subject A (a) and subject B (b). Each row represents one sample, and each column represents one iSNV position; invariant positions are not shown. iSNV frequencies are indicated by color. No chart is shown for subject C because no iSNVs were detected. Table S2 contains further information, including the exact position of each iSNV and its consensus and variant alleles.

Samples from subject A demonstrated the greatest diversity, with a total of 77 low-frequency iSNVs identified across the genome (see Table S2). Twenty-nine (38%) of the iSNVs were synonymous, and 48 (62%) were nonsynonymous. Fifteen (19%) were G-to-A changes in a dinucleotide context compatible with hypermutation by APOBEC3, a family of human proteins that play a role in antiviral innate immunity. The day 4 CVL sample had the highest number of iSNVs (*n* = 55) and the greatest average Shannon entropy (Fig. 6a); fewer iSNVs were present in day 4 plasma (*n* = 24) and even fewer at day 11 in CVL fluid (*n* = 5) and plasma (*n* = 6). These differences are unlikely to reflect iSNVs lost to low sequencing depth: all the samples had good depth of coverage (192× to 2,735×), and the sample with the lowest depth of coverage (day 4 CVL fluid) had the greatest number of iSNVs. Overall HIV-1 diversity therefore decreased between CVL fluid and plasma and between day 4 and day 11.

Upon closer examination of the iSNVs in subject A (Fig. 6a; see Table S2), several patterns emerged. Most of the iSNVs that were present in day 4 CVL fluid (52 out of 55) were at lower frequency or absent in day 4 plasma and were absent in both compartments by day 11. Most of these “bottlenecked” iSNVs were linked with at least one other iSNV within a 100-bp sequencing read at similar frequency (Table S2), suggesting that they might have been acquired from the transmitting partner. We also observed 17 iSNVs that were present only in the plasma at day 4 and in no other samples, including day 4 CVL fluid. Most of these were not linked with other iSNVs (*n* = 13; 76%), suggesting that they could have arisen during viral replication in either the plasma or regional lymph nodes prior to the development of viremia. The disappearance of all 69 of these iSNVs by day 11 in plasma suggests the presence of a bottleneck that restricted most of the HIV-1 quasispecies variants that were both transmitted and generated very early during infection.

A few iSNVs persisted between day 4 and day 11, while no new iSNVs arose during that week. One of the persistent iSNVs was present only in CVL fluid, and three were present only in plasma, suggesting possible compartmentalization. The remaining three persistent iSNVs were present in both compartments. While these persistent variants may have been transmitted, all were unlinked with other iSNVs, raising the possibility that they arose in the recipient partner prior to the detection of viremia. Each was present at similar frequencies between samples, suggesting that they may represent individual foci of infection in the FGT (being distributed through plasma) or elsewhere (seeding the FGT).

For subjects B and C, we observed substantially fewer iSNVs. In subject B, there were 14 iSNVs in the day 1 CVL sample (Fig. 6b; see Table S2), 9 (64%) of which were G-to-A changes compatible with hypermutation by APOBEC3. No iSNVs were identified in any other sample from this subject, which could in part be attributable to lower sequencing depth (due to low input levels of HIV-1 RNA), but also likely reflects some true loss of diversity. We used a binomial distribution to estimate the probability that iSNVs detected in CVL fluid on day 1 would not be detected in CVL fluid on day 7 (depth of coverage, 206×) due to chance. We found there would be a 0.05% probability of not detecting an iSNV with a frequency of 3.7% (the highest-frequency iSNV in day 1 CVL fluid) and a 16% probability of not detecting an iSNV with a frequency of 0.9% (the median frequency of iSNVs in day 1 CVL fluid, with 10 iSNVs in day 1 CVL fluid detected at this frequency or higher). It therefore seems unlikely that sequencing depth alone accounted for the lack of iSNVs in day 7 CVL fluid compared to day 1 CVL fluid, supporting a biological decrease in HIV-1 diversity between day 1 and day 7 in the FGT. We did not perform a similar comparison for plasma samples, because the sequencing depth was too low to make meaningful comparisons (mean depths of coverage, 37× for day 1 plasma and 50× for day 7 plasma).

In subject C, we did not identify any iSNVs in either CVL fluid or plasma at day 7, the earliest time point available to assess diversity (see Table S2). Both samples were sequenced to high depth (mean depths of coverage, 1,904× from day 7 plasma and 424× from day 7 CVL fluid), so our results likely reflect truly low HIV-1 diversity in both CVL fluid and plasma in this subject at day 7. These results are consistent with those for subject A, in whom very few iSNVs were detected by day 11, and subject B, in whom no iSNVs were detected at day 7.

### Within-sample HIV-1 complex variants.

**FIG 7:**
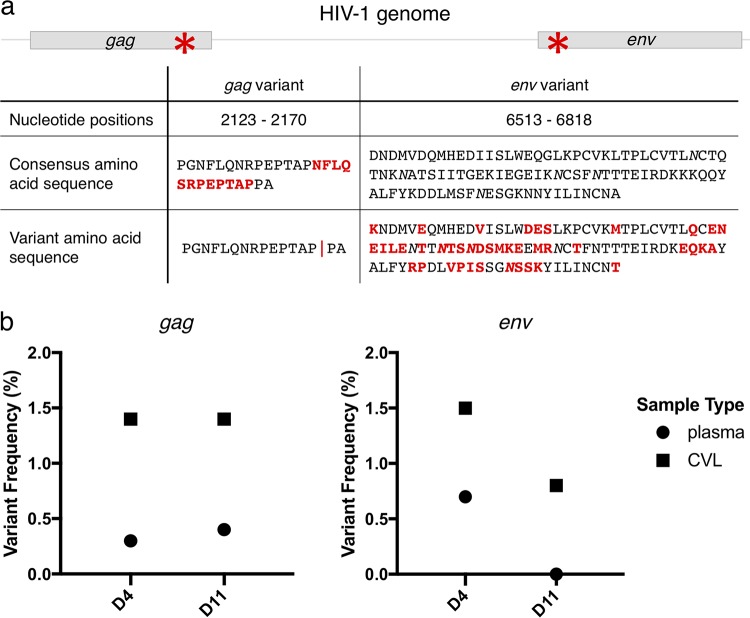
*gag* and *env* variants in subject A. (a) (Top) Schematic showing the genome positions of the deletion in the PTAP region of *gag* and the complex variant in *env* gp120 (prior to the V1 loop) in subject A, each marked with an asterisk. (Bottom) Nucleotide positions are indicated relative to the HXB2 reference sequence. Red indicates mismatches from subject A’s consensus sequence, and the red vertical line represents the 12-amino-acid deletion. The italicized *N*s indicate potential N-linked glycosylation sites. (b) Frequency of each variant in each sample from subject A. (Left) Frequencies of the *gag* deletion. (Right) Frequencies of the *env* variant. For *gag*, the plot shows the lower limit of the frequency of the deletion in each sample. Because the deletion occurred in an area of genome duplication, not all reads could be unambiguously mapped; the upper limit of the frequency of the deletion in each sample was approximately 25%. For *env*, all the reads could be unambiguously mapped, and the plot shows the frequency of the variant in each sample. Each sample is identified by the number of days (D) after the first positive HIV-1 fingerstick.

In addition to iSNVs, we sought to identify more complex intrahost variants by assembling reads *de novo* to capture regions of high diversity or insertions/deletions that would have been missed by our standard read mapping. We identified two complex variants in subject A, one in the *gag* gene and one in *env*. The *gag* variant, a 36-bp in-frame deletion (Fig. 7a), was detected in both plasma and CVL fluid at both time points and in both independent sequencing libraries from each sample, arguing against its being a PCR artifact. Its frequency, based on unique reads, was higher in CVL fluid than plasma at both time points (Fig. 7b, left). This deletion was found in the PTAP region of *gag*, which is known to harbor duplications, particularly in HIV-1 subtype C viruses (23). PTAP duplication has been associated with increased compensatory fitness in the setting of protease inhibitor resistance (24), which was likely not a contributing factor in this subject, because protease inhibitors are not commonly used in South Africa.

The *env* variant detected in subject A was located in gp120, outside the V1 loop on the 5′ end (Fig. 7a). This variant, 346 bp in length, shared only 75% nucleotide identity with the consensus HIV-1 sequence from subject A; it was equally different from the consensus sequences in subjects B and C (73% and 74% nucleotide identity, respectively). Using BLAST (NCBI), we found that the sequence was most similar (86% identity) to a subtype C HIV-1 isolate from South Africa (GenBank accession number AY463226.1). This was unlikely to be a contaminant, because we identified reads bridging this *env* variant with the consensus backbone on both the 5′ and 3′ ends, and other HIV-1 subtype C samples have not been previously sequenced in our laboratory. The *env* variant was present at low frequency overall, and similar to most of the iSNVs in subject A, it decreased in frequency between CVL fluid and plasma and between day 4 and day 11 (Fig. 7b, right). We did not identify any complex variants in subjects B and C.

### Metagenomic analysis.

**FIG 8:**
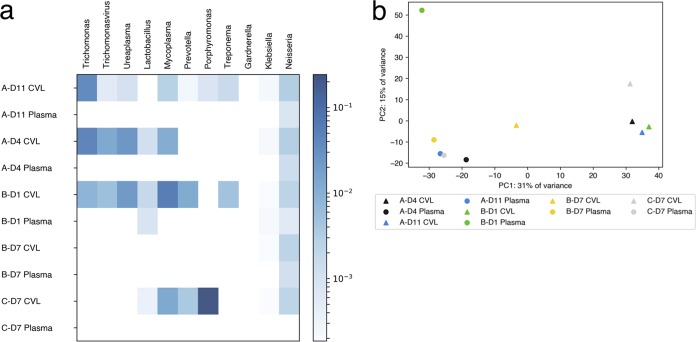
Metagenomic profiles of CVL fluid and plasma over time. (a) Results of metagenomic classification, indicating the log proportions of genera frequently found in CVL fluid after removal of human, HIV-1, and contaminating *Burkholderiales* reads. The columns represent genera, and the rows represent samples. Each sample is identified by subject identifier–number of days after the first positive HIV-1 fingerstick (D)–sample type. (b) PCA of centered log ratio-transformed genus abundance proportions for each sample.

We performed metagenomic classification of all nonhuman sequencing reads to characterize microbial diversity in the FGT. In addition to using water controls, we analyzed each CVL sample using its paired plasma sample as an internal control. CVL samples had overall increased microbial diversity compared to plasma, including a number of unique taxa that are commonly associated with the FGT environment (Fig. 8). These taxa, which were not detected in water controls, include commensal organisms, such as *Porphyromonas* and *Prevotella*, as well as organisms that may be commensal or associated with the disease state, such as *Ureaplasma* and *Mycoplasma*. Of note, our methods were not optimized to detect all bacterial species because we used CVL supernatants rather than cell pellets and we did not perform stringent lysis procedures, such as bead beating, that are necessary for some bacteria. Perhaps for these reasons, we did not detect high abundances of the genera *Prevotella*, *Gardnerella*, and *Gemella*, as had previously been detected in this cohort by 16S rRNA amplification and sequencing (18). Interestingly, the CVL sample from subject C on day 11 did not have high abundances of CVL fluid-associated taxa, and the sample clustered between plasma and CVL samples on principal-component analysis (PCA) (Fig. 8). The observed reduction in CVL fluid-associated taxa may be the result of administration of antibiotics (which was not noted at the study visit) or other interventions that would drastically reduce the microbial diversity in the FGT.

In some CVL samples, we detected reads from pathogens known to cause STIs, which were also not detected in our water controls. We detected *Trichomonas* and its associated bacteriophage in subjects A and B, who were also found to have *Trichomonas* by clinical STI screening. We detected Chlamydia trachomatis in subjects A and C, which had not been detected by clinical screening using nucleic acid amplification. Subject A was clinically diagnosed with Neisseria gonorrhoeae infection; although we detected reads from *Neisseria*, we were not able to definitively classify these as N. gonorrhoeae due to high rRNA sequence similarity among nonpathogenic species within the genus *Neisseria*. Furthermore, we consistently observed low levels of background reads from the genus *Neisseria* in water controls, as well as CVL and plasma samples from all three subjects (Fig. 8a). Therefore, our classification methods are not specific enough to confidently identify N. gonorrhoeae.

Overall, although our current methods were not optimized for sequencing all bacteria, we did detect known representatives of the FGT microbiome and STI, providing proof of concept for the use of metagenomic sequencing in evaluating complex sites, such as the FGT, that contain viral and protozoan populations, which would not be detected by bacterial 16S rRNA sequencing.

## DISCUSSION

Overall, we found little diversity in the HIV-1 RNA quasispecies populations in both the FGT (CVL samples) and plasma in three women with very early HIV-1 infection. Our findings are compatible with a very early HIV-1 transmission bottleneck, which was mostly complete by the time viremia was detectable in these three individuals. Because our study design did not include samples from the transmitting partners, we cannot exclude the possibility that the subjects were exposed to a relatively homogeneous viral population (e.g., if the transmitting partners had acute infections themselves). We also were not able to assess contributions to the HIV-1 transmission bottleneck prior to the FGT or assess features of transmitted viruses compared to nontransmitted viruses, as in prior studies that included transmission pairs (1, 3).

Nevertheless, by comparing HIV-1 populations in the FGT—the site of HIV-1 acquisition—and blood compartments over the first 1 to 2 weeks after viremia was detectable, we observed a reduction in HIV-1 diversity suggestive of a bottleneck during hyperacute infection. This was most evident in subject A, who had clearly demonstrable low-frequency HIV-1 quasispecies diversity in the FGT sampled 4 days after detection of viremia, including multiple sets of iSNVs linked on 100-bp sequencing reads and a complex 300-bp *env* variant. Because it would be unlikely for multiple mutations to occur so closely together on the same HIV-1 template this early in infection, many of these variants were likely acquired from the transmitting partner. The complex *env* variant was quite divergent from the consensus *env* sequence, but reads were present that bridged it to the consensus backbone on both sides, arguing against contamination. We cannot exclude the possibility that this variant was transmitted by a different partner in close temporal proximity and subsequently recombined with the consensus variant. The disappearance of the *env* variant and other sets of linked iSNVs in the plasma by day 11 supports the presence of a bottleneck between the FGT and blood at this very early time in infection. However, the bottleneck was not absolute; in subject A, three unlinked iSNVs and a *gag* deletion persisted at a frequency of 1% to 5% of the HIV-1 population in both compartments through day 11. We therefore characterize subject A as having multiple transmitted viruses (detected in day 4 CVL fluid) and a smaller number of founder viruses (detected in day 11 CVL fluid and plasma). It remains unclear whether the low-frequency founder viruses, which contained unlinked iSNVs, were transmitted or arose through replication in the FGT during the eclipse phase prior to the development of viremia.

In subject B, we observed HIV-1 quasispecies diversity in the FGT at day 1, but not at day 7 or in plasma at either time point, though these samples had lower HIV-1 loads and consequently lower depth of coverage in sequencing. In subject C, we observed no HIV-1 quasispecies diversity in the FGT or blood 1 week after the detection of viremia. We therefore characterize subjects B and C as having single founder viruses. Interestingly, all three women in our study had little HIV-1 diversity despite using hormonal contraceptives and despite the presence of concurrent STIs; these factors have been associated with acquisition of a more diverse HIV-1 population in prior studies (12, 13).

Our study was limited by the inclusion of only three individuals. Notably, however, the FRESH study design uniquely enabled us to investigate samples from the blood and FGT within the first 2 weeks after viremia was detectable. Prior studies of the HIV-1 transmission bottleneck have examined samples several months after infection, mostly from blood. Recently, Klein et al. observed a greater number of distinct HIV-1 *env* C2-V3-C3 clones present in the FGT than in blood in 12 women with matched cervical and plasma samples collected between 0 and 3 months after infection (10). Similarly, we found greater HIV-1 diversity in FGT than in blood in two out of three subjects for at least one time point. Interestingly, however, Klein et al. reported that the predominant HIV-1 clone in the FGT was most often different from the predominant HIV-1 clone in blood, whereas we found the same HIV-1 consensus sequence in FGT and blood in all three subjects and at all the time points examined. It is possible that by chance we investigated three subjects who did not acquire as diverse an HIV-1 population as the subjects in the earlier study. The difference could also be explained by compartmentalized evolution between the time of infection and sampling, with a shorter period available for compartmentalized evolution in our study.

Our results are consistent with prior animal studies, in which macaques vaginally infected with a diverse simian immunodeficiency virus (SIV) population were found to have a reduced number of viral variants within weeks after inoculation, both in the plasma (25, 26) and in the vaginal tract (27). Given the challenge of capturing similarly early time points in human studies, our study represents the earliest examination of HIV-1 quasispecies in the human FGT, to our knowledge. Our results suggest that future work is needed to investigate viral diversity within the FGT even earlier in infection, ideally including FGT samples collected prior to peak viral load in the FGT and prior to the development of viremia, with comparison to the transmitting partner samples.

Our sequencing and analytic methods offer several advances for the field of HIV-1 genome sequencing. Metagenomic sequencing has previously been employed to assemble consensus HIV-1 genomes (28, 29), and here, we expand the use of this technique to quantify iSNVs from high-depth next-generation sequencing (NGS) in HIV-1 infection. Historically, HIV-1 quasispecies diversity has been assessed by endpoint dilution to single-genome templates, which limits the number of templates that can be assessed, or, alternatively, by PCR amplification followed by NGS, which can be limited by amplification bias, recombination, and resampling. Our sequencing libraries are randomly generated so that each read that is derived from a unique template has a unique start position and end position, allowing removal of the duplicates that are generated by limited-cycle PCR. This approach is conceptually similar to primer ID (30) but allows identification of unique reads based on characteristics of the starting and ending positions of the reads themselves.

An additional benefit of this approach is the opportunity to perform metagenomic analysis of other organisms in a sample, which is especially important in HIV-1 transmission at microbially diverse sites, such as the FGT. Although our current methods were not optimized for sequencing bacteria, we did detect known representatives of the FGT microbiome, including organisms causing sexually transmitted infections. A more comprehensive evaluation of bacterial species could be achieved with the addition of upstream processing steps, e.g., bead beating for bacterial lysis. Unlike bacterial 16S rRNA sequencing, metagenomic sequencing offers the potential to interrogate a wide range of microbes, including protozoa and viruses. This technique, therefore, has broad application to the study of HIV-1, including viral population diversity and coinfections.

In conclusion, we utilized metagenomic sequencing to study HIV-1 populations present in the blood and FGT during the earliest stages of acute infection and observed little viral diversity, supporting a very early transmission bottleneck.

## MATERIALS AND METHODS

### Ethics statement.

The study protocol was approved by the Biomedical Research Ethics Committee of the University of KwaZulu-Natal and the Partners Institutional Review Board (IRB) (2012P001812/MGH). The Broad Institute of MIT and Harvard has a standing reliance agreement with Partners through which it relied on the Partners IRB to provide a review of this study. Written informed consent was obtained from all participants following the explanation of the nature and possible consequences of the study; all the participants were 18 years of age or older.

### Study cohort.

The women were enrolled in the FRESH study, a prospective observational study conducted near Durban, South Africa (19). The participants received HIV-1 infection prevention counseling, and both female and male condoms were provided at the study site. To be eligible for the study, participants had to be female, 18 to 23 years old, HIV-1 uninfected, and sexually active. They further had to be willing to adhere to study requirements, to have HIV-1 tests performed twice per week, and to have samples stored. Exclusion criteria included pregnancy, anemia, enrollment in another study, and engagement in full-time employment or school. None of the subjects included had a known history of injection drug use.

### Clinical procedures.

Twice per week, the participants attended classes focused on HIV-1 infection prevention, personal empowerment, and job skills training. At each visit, they underwent a finger prick blood draw for quantitative HIV-1 RNA testing. Every 3 months, the participants had a peripheral blood draw and pelvic examination (not performed during menstruation) that included the collection of CVL fluid for sampling from the FGT. CVL fluid was obtained by washing the cervicovaginal walls with 5 ml of sterile saline, which was then centrifuged at 1,700 rpm at 4°C to pellet cells, and the cell-free supernatant, containing free viral particles, was used for RNA sequencing and further analysis in the study (18–20). The participants also completed a detailed HIV-1 risk questionnaire, which was administered by a counselor; it included STI history, sexual behavior, family planning, use of antibiotics, and diet. Upon detection of a positive HIV-1 RNA test result, the participants underwent blood collection and pelvic examinations with CVL sample collection at 1, 2, 3, 5, 9, 12, 24, 36, and 48 weeks postdetection.

### Measurement of HIV-1 load in CVL fluid and plasma.

HIV-1 clinical viral load testing in plasma was performed by the Global Clinical Viral Laboratory, South Africa, as previously described (18). Viral RNA was extracted from 500 to 1,000 μl of plasma and 500 μl of CVL supernatant using a QIAamp Viral RNA Mini Kit (Qiagen) according to the manufacturer’s instructions, including a step for on-column DNase. HIV-1 RNA was quantified by one-step quantitative reverse transcription (qRT)-PCR using a QuantiFast SYBR Green RT-PCR kit (Qiagen) and the following *gag* primers (from the Amplicor HIV-1 Monitor viral load test): SK145 primer (forward), AGTGGGGGGACATCAAGCAGCCATGCAAAT; SK431 primer (reverse), TGCTATGTCACTTCCCCTTGGTTCTCT (IDT). PCR conditions were as follows: (i) RT, 50°C for 10 min; (ii) reactivation, 95°C for 5 min; (iii) 40 cycles of 95°C for 10 s and 60°C for 30 s; (iv) melting curve, 95°C for 15 s, 60°C for 15 s, and then ramp to 95°C; (v) cooling, 40°C for 30 s. Precise calculation of viral copy numbers was achieved by using a standard curve derived from a linear, nearly full-length plasmid HIV-1B genome fragment that had been prepared by digestion of an HIV-1B infectious molecular clone (pNL4-3 and pHXB2-RU3), gel purification, and quantification by spectrophotometry (Nanodrop; Qubit).

### Metagenomic sequencing.

Sequencing libraries were constructed from 5 μl of RNA, corresponding to a starting input of at least 25,000 HIV-1 copies (and in most cases at least 250,000 HIV-1 copies). The library construction methods have been previously described (31, 32). Briefly, carrier RNA [poly(rA)] that had been introduced during the RNA extraction process was depleted using 40-nucleotide (nt) oligo(dT) probes and Hybridase thermostable RNase H (Epicentre), followed by RNase-free DNase treatment (Qiagen). cDNA was constructed using random-hexamer primers and SuperScript III (Invitrogen) for first-strand synthesis, followed by second-strand synthesis (NEB). There is no amplification during this step. Sequencing libraries were generated using the Nextera XT DNA Library Prep kit (Illumina), which uses transposases to randomly fragment the cDNA, resulting in unique start and end positions of the cDNA fragments prior to any amplification steps. Sequencing adapters including dual indexes were added, and the cDNA fragments underwent amplification with 16 cycles of PCR. The libraries were quantified using a KAPA universal complete kit (Roche), pooled to equal concentration with 4 to 10 samples per lane, and sequenced on an Illumina MiSeq or HiSeq using paired-end 101-bp reads. As a negative control, a water sample was included with each batch of library construction and sequencing. Duplicate independent sequencing libraries were made from each RNA sample. Reads from duplicate libraries were merged and analyzed together to assemble consensus HIV-1 genome sequences. Duplicate libraries were also analyzed independently to verify the presence of iSNVs, as described below.

### Metagenomic analysis.

Reads were taxonomically classified using a combination of BWA (42), Kraken (43), and DIAMOND (44). The first-pass classification was via bwa mem v0.7.15 with a custom database containing SILVA (45) LSU and SSU Ref rRNA sequences (release 128), the whole human genome (hg38), and all the sequences from NCBI’s viral accession list (46) with a human host as of October 2015. PCR and optical duplicates were removed via Picard MarkDuplicates v2.6.0. The remaining reads were further classified with DIAMOND v0.8.18.80 on NCBI’s *nr* database. Lastly, reads were classified using Kraken v0.10.6 on a custom database built on the default “full” database containing all RefSeq whole genomes of bacteria and viruses as of October 2015. All Kraken classified reads were additionally subjected to a kraken-filter step with a threshold of 0.05 to reduce false-positive hits. Sequences added to the “full” Kraken database include all whole chromosomes from PlasmoDB (47) and RefSeq (48) whole genomes of fungi, protozoa, and plasmids (October 2015). Reads that had hits to multiple taxa were assigned to the hits’ lowest common ancestor taxon. Taxa that had <0.01% cumulative read abundance had their reads pushed up the taxonomy tree until all nodes contained at least 0.01% read abundance.

Given the high sensitivity and unbiased nature of metagenomic sequencing, we and others (33–36) frequently detect microbial reads that are present as background in reagents or the laboratory environment. Failure to account for this background can lead to erroneous identification of pathogenic microbes, as recently described (37). For this study, we addressed the presence of background microbial reads in two ways. First, we included a water sample as a negative control with each sequencing batch, which went through the entire library construction and sequencing process. Microbial species that we commonly detect in water controls include the genera *Burkholderia*, *Ralstonia*, *Cupriavidus*, and *Pseudomonas*. Second, we analyzed each CVL sample using its paired plasma sample as an internal control.

### HIV-1 genome assembly.

HIV-1 genomes were assembled using a published and freely available pipeline called viral-ngs (38), described in detail at https://viral-ngs.readthedocs.io. Briefly, samples were demultiplexed, and reads from human and known laboratory microbial contaminants (e.g., Escherichia coli and Pseudomonas fluorescens) were removed. For each sample, reads from duplicate independent sequencing libraries were combined. The consensus HIV-1 genome sequence of the viral population from each sample was constructed by *de novo* assembly using reads that matched a database of HIV-1 reference genomes (GenBank accession numbers are provided in File S1 in the supplemental material). Assemblies were completed by scaffolding *de novo* contigs against a subtype C reference genome (GenBank accession no. AF286227), followed by two rounds of refinement with the unfiltered reads. Consensus sequences represent the full HIV-1 genome, excluding long terminal repeats (LTRs), which could not be unambiguously assembled using this method.

To calculate the depth of sequencing and analyze within-sample variants, all HIV-1 reads from a sample were mapped to the consensus HIV-1 genome from that sample using Novoalign (Novocraft). Because cDNA fragments generated by Nextera XT library preparation have unique start and end positions, all reads with the same start and end positions were collapsed to their consensus, allowing removal of PCR duplicates and correction for errors generated during PCR and sequencing. The reported HIV-1 genome coverage is based on unique (deduplicated) reads.

### Phylogenetic analysis.

Consensus genome sequences were aligned to reference sequences using Geneious 8.1.7 (Biomatters). Regions that could not be unambiguously aligned were manually removed. A maximum-likelihood tree was constructed using PhyML (39) with automatic model selection (GTR) and 1,000 bootstrap replicates.

### Identification and analysis of iSNVs.

iSNVs were identified using V-Phaser2 (22). To distinguish true iSNVs from sequencing errors, iSNVs were restricted to those present in two independent sequencing libraries and in at least one forward and one reverse read and with forward or reverse strand bias of 5-fold or less. We also restricted our analysis to iSNVs present at 0.5% frequency or greater, since lower-frequency iSNVs could not be confidently identified with the above-mentioned criteria and the sequencing depth achieved for these samples. Sites meeting these criteria were manually inspected and removed from the final iSNV list if their positions in reads did not appear to be evenly distributed across the read length (40). For each sample, Shannon entropy was calculated as the sum across all iSNVs of negative ln(frequency) times frequency and then was divided by the total sequence length to yield the average Shannon entropy.

### Validation of the iSNV identification method.

To validate the method described above for identifying iSNVs, metagenomic sequencing was performed from RNA from a plasma sample from a patient with Feibig stage IV infection in a different cohort. This sample had previously undergone SGA and 454 sequencing of the 5′ half of the genome, generating a total of 19 sequences, among which 49 iSNVs were identified (2). Metagenomic sequencing was performed as described above to a moderate depth (45×) in order to compare iSNV detection between the two methods. For each of the 5 iSNVs identified by SGA but not by metagenomic sequencing, we manually inspected the metagenomic sequencing reads.

To assess whether the differences between the two methods were more than could be expected from sampling variance, we applied Fisher’s exact test to each iSNV, including those that failed the duplicate library and strand bias filters. To better understand the resulting *P* value distribution, we simulated the comparison between the two methods. SGA and metagenomic sequencing results were both modeled as binomial random variates, based on allele frequency. A set of 49 samples was simulated, with coverage and allele frequencies taken from the observed data (the allele frequency was estimated as the mean of those measured by the two methods); the coverage for the two metagenomic libraries was also taken from the data. Metagenomic sequencing had two filters imposed: both alleles had to be seen in two libraries, and both had to be seen on at least one forward and one reverse read. Forward and reverse strand assignment was modeled as a binomial random variate with no strand bias.

### Identification and analysis of complex intrahost variants.

In addition to a full-length consensus genome, viral-ngs also assembled subgenomic contigs using Trinity (version 2011-11-16), a transcriptome assembly program that can produce alternative contigs often used to identify splice isoforms in eukaryotic transcripts. This analysis allowed detection of complex variants that would have been missed in the initial mapping of HIV-1 reads to the consensus genome from each sample, either due to the presence of insertions/deletions or due to high diversity that would have been excluded by our Novoalign (Novocraft) parameters (-r Random -l 40 -g 40 -x 20 -t 1,500 -k). To assess the frequency of each variant identified, all the sequencing reads were negatively filtered for human and known laboratory contaminants, and these reads were aligned independently to the consensus and variant genomes. The variant frequency was defined as the percentage of reads that mapped to the variant genome unambiguously.

### Accession number(s).

The HIV-1 consensus genome sequences are available under GenBank accession numbers MH933704 to MH933714, and all the metagenomic sequencing reads (cleaned of human reads) are available on NCBI under BioProject PRJNA473698.

## Supplementary Material

Supplemental file 1

Supplemental file 2

Supplemental file 3
